# Training and Implementation Challenges in Adolescent Group Therapy within Public Mental Health Services

**DOI:** 10.1192/j.eurpsy.2026.11141

**Published:** 2026-07-31

**Authors:** L. López Gómez-Miguel, A. Gutiérrez Fernández de Velasco, Á. Privado Aranda, P. Bang Carrascosa, J. García Iglesias, M. González San José, M. Rodriguez Fernández, M. I. Montañés Rodríguez, A. Ladera de la Oliva, A. S. Benito Martin-Preciados, L. M. F. Oliva, M. Andrade Arango, E. García Martín de la Fuente, R. C. Ramos, M. D. R. D. F. Rodríguez, L. P. Ayuso Morales

**Affiliations:** 1 Hospital Universitario de Toledo, Toledo, Spain; 2 Programa de Transición y Primeros Consumos; 3 Programa de Transición a la vida adulta y Primeros consumos, Hospital Universitario de Toledo, Toledo, Spain; 4 Clínica Nuestra Señora de La Paz, Bogotá, Colombia

## Abstract

**Introduction:**

Despite strong evidence for group psychotherapy, its implementation in adolescent mental health services remains limited. Barriers include lack of professional training, insufficient supervision, and rigid adherence to specific theoretical schools.

**Objectives:**

- To identify barriers in the training and implementation of adolescent group therapy.
- To present insights from the Toledo Transition Program on overcoming these barriers.
- To propose recommendations for professional development and service design.

**Methods:**

Qualitative descriptive study based on clinical experience. Observation of group sessions, supervision dynamics, and training needs of psychiatry residents and staff were analyzed.

**Results:**

- Residents were often relegated to observer roles in groups, limiting active learning.
- Lack of supervision spaces hindered skill acquisition and confidence.
- Rigid theoretical frameworks discouraged flexible, integrative approaches.
- Program experience showed that transdisciplinary teamwork and integrative use of dynamic, systemic, and cognitive tools improved training outcomes.

**Conclusions:**

Expanding group therapy training requires supervision, active participation, and theoretical flexibility. Addressing these gaps can strengthen workforce capacity and facilitate the integration of group interventions as a standard tool in adolescent psychiatry.

**Disclosure of Interest:**

None Declared

